# Anti-Inflammatory Activity of Orally Administered *Monostroma nitidum* Rhamnan Sulfate against Lipopolysaccharide-Induced Damage to Mouse Organs and Vascular Endothelium

**DOI:** 10.3390/md20020121

**Published:** 2022-02-03

**Authors:** Masahiro Terasawa, Keiichi Hiramoto, Ryota Uchida, Koji Suzuki

**Affiliations:** 1 Faculty of Pharmaceutical Sciences, Suzuka University of Medical Science, Minamitamagaki-cho 3500-3, Suzuka 513-8670, Mie, Japan; terasawa@konanchemical.co.jp (M.T.); hiramoto@suzuka-u.ac.jp (K.H.); uchida@konanchemical.co.jp (R.U.); 2 Konan Chemical Manufacturing, Co., Ltd., 1515 Kitagomizuka, Yokkaichi 510-0103, Mie, Japan

**Keywords:** *Monostroma nitidum*, rhamnan sulfate, endothelium, anti-inflammation, glycocalyx

## Abstract

We previously reported that rhamnan sulfate (RS) purified from *Monostroma nitidum* significantly suppressed lipopolysaccharide (LPS)-induced inflammation in cultured human vascular endothelial cells. Here, we analyzed the effect of orally administered RS on LPS-induced damage to mouse organs and vascular endothelium. RS (1 mg) was orally administered daily to BALB/c mice, 50 μg of LPS was intraperitoneally administered on day 8, and Evans blue was injected into the tail vein 6 h later. After 30 min, LPS-treated mice showed pulmonary Evans blue leakage and elevated plasma levels of liver damage markers, whereas this reaction was suppressed in LPS + RS-treated mice. Immunohistochemical and Western blot analysis of mouse organs 24 h after LPS treatment showed significant neutrophil infiltration into the lung, liver, and jejunum tissues of LPS-treated mice and high expression levels of inflammation-related factors in these tissues. Expression levels of these factors were significantly suppressed in LPS + RS-treated mice. Analysis of lung glycocalyx showed a significant reduction in glycocalyx in LPS-treated mice but not in LPS + RS-treated mice. Levels of syndecan-4, one of the glycocalyx components, decreased in LPS-treated mice and increased in LPS + RS-treated mice. The current results suggest that orally administered RS protects organs and vascular endothelium from LPS-induced inflammation and maintains blood circulation.

## 1. Introduction

**Figure 1:**
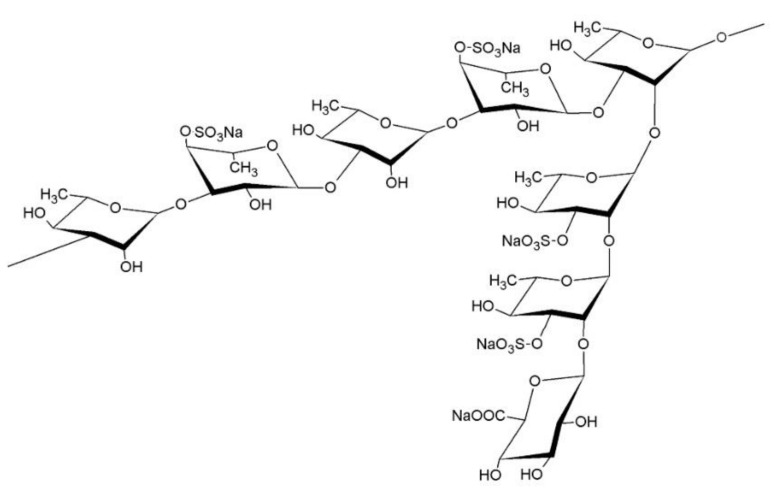
Chemical structure of rhamnan sulfate (RS). RS is composed of linear chains of L-rhamnose (α-1,3 linkages) with branched chains (α-1,2 linkages), to which several sulfate groups are bound; reproduced from Tako et al. [2].

Seaweed is traditionally consumed by people living in coastal areas of East Asia. It is rich in vitamins and minerals and is known to contain polysaccharides that have a positive effect on the human body. *Monostroma (M.) nitidum* is a green alga that is composed of many cell aggregates containing intercellular substances that fill the spaces between cells. These intercellular substances are rich in dietary fiber, vitamins, and minerals. Rhamnan sulfate (RS) is a soluble dietary fiber of intercellular substances. RS is composed of straight and branched chains of rhamnose units, and approximately 25% of these have sulfate groups (Figure 1) [1,2,3,4]. RS has been reported to have anticoagulant and antiviral activities in herpes, influenza, SARS-Co-V2, etc., and cholesterol-lowering effects [1,5,6,7,8,9,10,11,12,13]. In addition, clinical studies have shown that RS has a constipation-preventing effect by altering the gut microbiota [14]. These results indicate that RS plays an important role in the health-promoting effects of *M. nitidum*.

Activation of blood coagulation and inflammation of the vascular endothelium are closely associated with the onset of thrombosis. Inflammation of the vascular endothelium causes pathological conditions such as hypertension, arteriosclerosis, arterial thrombosis, and venous thrombosis [15,16]. Monocytes and macrophages accumulate in inflammatory vascular endothelial cells. Tissue factor (TF) expressed on these cells causes extrinsic blood coagulation [17,18]. A von Willebrand factor (VWF) is produced and secreted by inflammatory vascular endothelial cells and stimulates platelet aggregation [19]. Lipopolysaccharide (LPS), which is present in the cell membrane of Gram-negative bacterial, binds to Toll-like receptor 4 and stimulates the inflammatory response of animal cells. LPS promotes the production of TF and VWF by vascular endothelial cells [8,19], resulting in the promotion of blood coagulation and platelet aggregation. Additionally, it induces the secretion of inflammatory cytokines such as interleukin (IL)-6 and tumor necrosis factor (TNF)-α by vascular endothelial cells and the expression of various cell adhesion molecules, such as E-selectin and the vascular cell adhesion molecule (VCAM)-1 [20]. It also promotes the migration of leukocytes to the site of inflammation, which further enhances inflammation [21,22]. Therefore, suppressing inflammation of vascular endothelial cells is very important for preventing thrombus formation in blood vessels.

Glycocalyx, present on the surface of animal cells, protects living cells and organ tissues from a variety of physical and chemical disorders. It is present on the surface of vascular endothelial cells and is estimated to be approximately 500 nm thick [23]. The glycocalyx coating of vascular endothelial cells is involved in the regulation of vascular barrier function and circulating blood volume, smooth movement of blood cells in the vessels, function of vascular smooth muscle via nitric oxide, protection of vascular endothelial cells, and regulation of inflammatory reaction and coagulation ability [24]. Glycocalyx is composed of proteoglycans, glycoproteins, and sulfated sugar chains, such as heparan sulfate and chondroitin sulfate [25]. Glycocalyx regulates the binding of leukocytes, platelets, and coagulation/inflammation-related factors to other substances in the blood under normal conditions. The LPS-induced inflammatory response induces glycocalyx shedding and inflammation by causing leukocytes to bind to the vascular endothelium [26,27]. When animals are treated with LPS, the glycocalyx on the vascular endothelium is removed, and inflammation of the vascular endothelium is induced [28].

Syndecan, a component of glycocalyx, is a representative transmembrane proteoglycan that binds to the cytoskeleton. There are four subtypes of syndecan, of which syndecan-1, -2, and -4 are expressed on endothelial cells. Syndecan-4, which contains heparan sulfate, plays an important role in the regulation of blood coagulation because it specifically binds to antithrombin and greatly enhances its anticoagulant activity [26]. Syndecan-4 deficiency increases the mortality rate of mice injected with LPS [29]. 

Antithrombin has anticoagulant and anti-inflammatory properties. Antithrombin binds to the heparan sulfate of syndecan-4 and stimulates endothelial cells to produce prostacyclin, which suppresses the adhesion of neutrophils and platelets to endothelial cells and suppresses endothelial inflammation [30]. This is because syndecan-4 has pentasaccharide arrays, which are recognized as heparin-binding sites by antithrombin [31]. Therefore, antithrombin bound to syndecan-4 is important to protect the structure of the glycocalyx coating on vessel walls during inflammation [32,33]. 

We have previously reported that RS, like heparin, enhances the thrombin inhibitory activity of antithrombin, and, unlike heparin, has a significant anti-inflammatory effect on LPS, TNF-α, and thrombin-induced cultured human endothelial cells [8]. However, it is unclear whether RS exhibits anti-inflammatory properties in vivo. Therefore, in this study, we investigated the effect of oral administration of RS on LPS-induced inflammation in mice. We found that RS allowed the maintenance of blood circulation and protected mouse organs and vascular endothelium from LPS-induced inflammation.

## 2. Results

### 2.1. Effect of RS on LPS-Induced Lung Vascular Leakage

**Figure 2:**
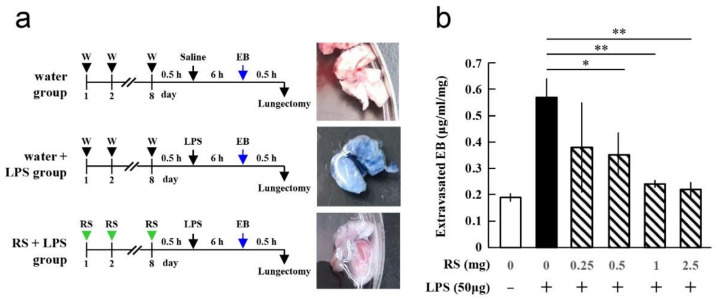
Effects of oral daily administration of rhamnan sulfate (RS) or water on lipopolysaccharide (LPS)-induced pulmonary vascular permeability in mice. (**a**) Experimental schedules of RS, LPS, and Evans blue (EB) administration and lung sampling. Water (W) or varying doses of RS (0.25, 0.5, 1, or 2.5 mg) were orally administered daily for 8 d. On day 8, mice were intraperitoneally treated with LPS (50 μg) or saline 30 min after last water or RS administration. Six hours later, EB was injected into the tail vein. Lungs were resected 30 min after EB injection. Pictures of the lungs resected from each group are shown on the right. (**b**) Effects of different doses of RS on LPS-induced EB extravasation. The amount of extravasated EB was calculated by the method shown in Materials and Methods. The data are shown as mean ± standard deviation (SD). * *p* < 0.05 and ** *p* < 0.01 indicate significant differences between water group (RS: 0, LPS: -), water + LPS group (RS: 0, LPS: +), and RS+LPS group (RS: 0.25, 0.5, 1, or 2.5 mg/mouse and LPS: +) by Tukey’s multiple comparisons test (*n* = 3).

LPS induces an inflammatory response in animal organs, including increased vascular permeability [34]. The effect of RS on pulmonary vascular permeability in LPS-induced sepsis in mice was first investigated. Increased pulmonary vascular permeability is a typical pathological feature of sepsis. After oral administration of water or different doses of RS daily for 8 d, LPS was intraperitoneally administered (water + LPS group or RS + LPS group) (Figure 2a). Only water was administered for 8 d to the control group (water group). Six hours after LPS treatment, Evans blue (EB) dye was injected into the tail vein of mice to assess extravasation of the dye into the lungs. As shown in the mouse lung photographs, significant leakage of EB was observed in the lung tissue of the water + LPS group compared to the control water group, and EB leakage was suppressed in the RS + LPS group. The data indicated that the significant increase in pulmonary vascular permeability due to LPS was suppressed by prior oral administration of RS. Figure 2b shows the effect of the administration of different doses of RS prior to LPS treatment on the amount of extravasation of EB from the lungs of mice. Administration of RS prior to LPS treatment suppressed the increase in LPS-induced EB extravasation from the lung in a dose-dependent manner. This indicates that oral administration of >1 mg of RS per mouse protects against LPS-induced vascular permeability of the lungs during inflammation.

### 2.2. Effect of RS on LPS-Induced Muscle and Liver Damages

**Figure 3:**
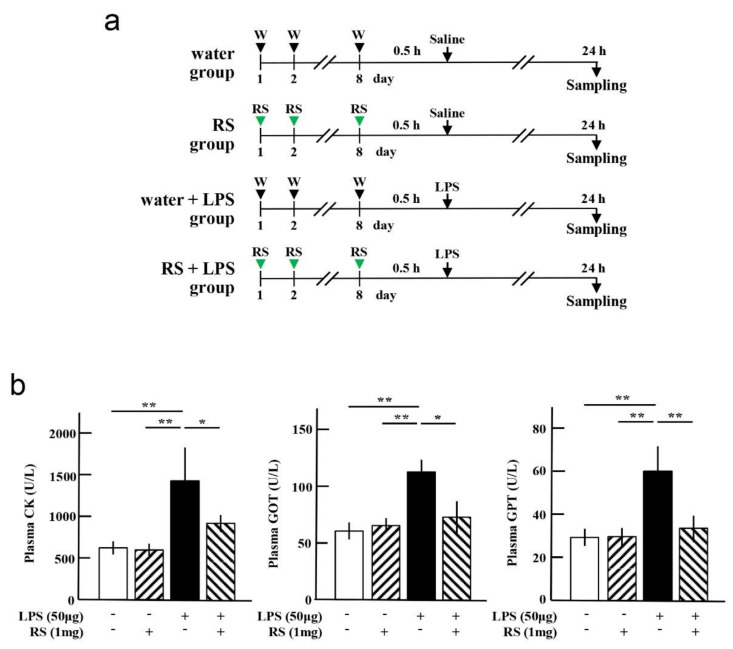
Effects of oral daily administration of RS or water on mouse organs after treatment with LPS or saline. (**a**) Experimental schedules for investigation of the effect of oral administration of RS (1 mg) or water on the organ tissue of mice 24 h after treatment with LPS (50 μg) or saline in the water group (RS: -, LPS: -), RS group (RS: +, LPS: -), water + LPS group (RS: -, LPS: +), and RS+LPS group (RS: +, LPS: +). (**b**) Plasma levels of creatine kinase (CK), glutamic oxaloacetic transaminase (GOT), and glutamic pyruvic transaminase (GPT) in each group of mice. The data are shown as mean ± SD. * *p* < 0.05 and ** *p* < 0.01 indicate significant differences between the respective groups by Tukey’s multiple comparisons test (*n* = 5).

Next, the effect of oral RS administration on plasma and expression levels of marker proteins indicating organ damage induced by LPS treatment was investigated. The experimental procedure is illustrated in Figure 3a. Water or 1 mg of RS was orally administered daily for 8 d, followed by intraperitoneal injection of LPS (water + LPS group or RS + LPS group). Water or RS was administered for 8 d to the control group, followed by an intraperitoneal injection of saline (water group and RS group). Twenty-four hours post-LPS treatment, heparinized plasma samples were collected from the hearts of the mice, and several organs were removed from the mice.

Plasma levels of creatine kinase (CK), markers of muscle damage, and glutamic pyruvic transaminase (GPT) and glutamic oxaloacetic transaminase (GOT), markers of hepatocellular injury, were all significantly increased in mice of the water + LPS group (*p* < 0.01), and these increases were significantly suppressed in mice of the RS + LPS group (*p* < 0.05 or *p* < 0.01) (Figure 3b). These findings indicate that oral administration of RS suppresses LPS-induced damage to muscle and hepatocytes.

### 2.3. Effect of RS on LPS-Induced Morphological Changes in Various Organs

**Figure 4:**
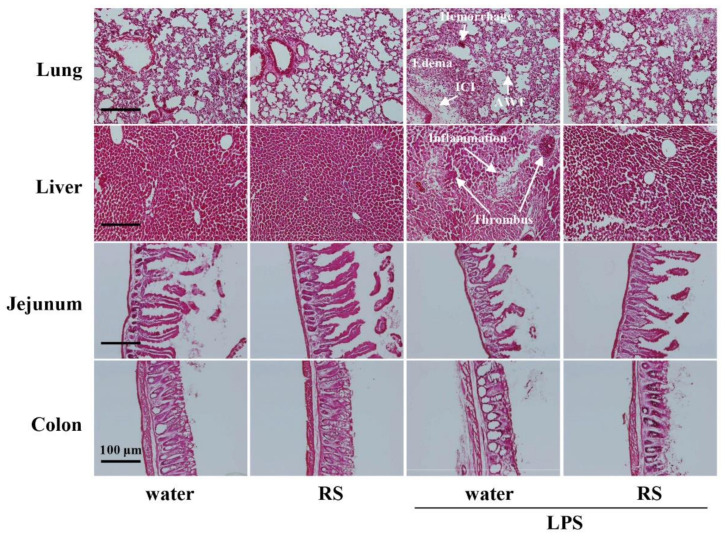
Effects of oral daily administration of RS (1mg) or water on morphological changes in organs of mice 24 h after treatment with LPS (50 μg) or saline. Hematoxylin and eosin staining of organ tissues of mice in the water group (RS: -, LPS: -), RS group (RS: +, LPS: -), water + LPS group (RS: -, LPS: +), and RS + LPS group (RS: +, LPS: +). Scale bars: 100 μm. Arrows show alveolar hemorrhage, alveolar wall thickening (AWT), and inflammatory cell infiltration (ICI) in the lungs, and erosive inflammation and thrombus in the liver.

Histopathological analysis of hematoxylin–eosin (HE)-stained sections from mouse lungs, liver, jejunum, and colon were performed to investigate the effects of RS on LPS-induced organ damage. As shown in Figure 4, histopathological changes were observed in all organs of mice in the water + LPS group, whereas no change was observed in the organs of the water group (normal). In the lungs, edema, alveolar hemorrhage, alveolar wall thickening, and inflammatory cell infiltration were observed in mice of the water + LPS group. These changes were reduced in the lungs of the RS+LPS group. In the liver, erosive inflammation and thrombus formation were observed in mice of the water + LPS group, and lesion tissue decreased in the RS+LPS group. In addition, liver cell density was clearly increased in mice of the water+RS group. In the jejunum, microvilli size decreased in mice of the water + LPS group, but this decrease was suppressed in the RS+LPS group. In the colon, vacuoles were induced in mice of the water + LPS group but decreased in the RS + LPS group. The data indicate that prior RS administration suppresses inflammatory morphological changes in the lungs, liver, jejunum, and colon induced by LPS treatment.

### 2.4. Effect of RS on LPS-Induced Infiltration of Neutrophils in Organs

**Figure 5:**
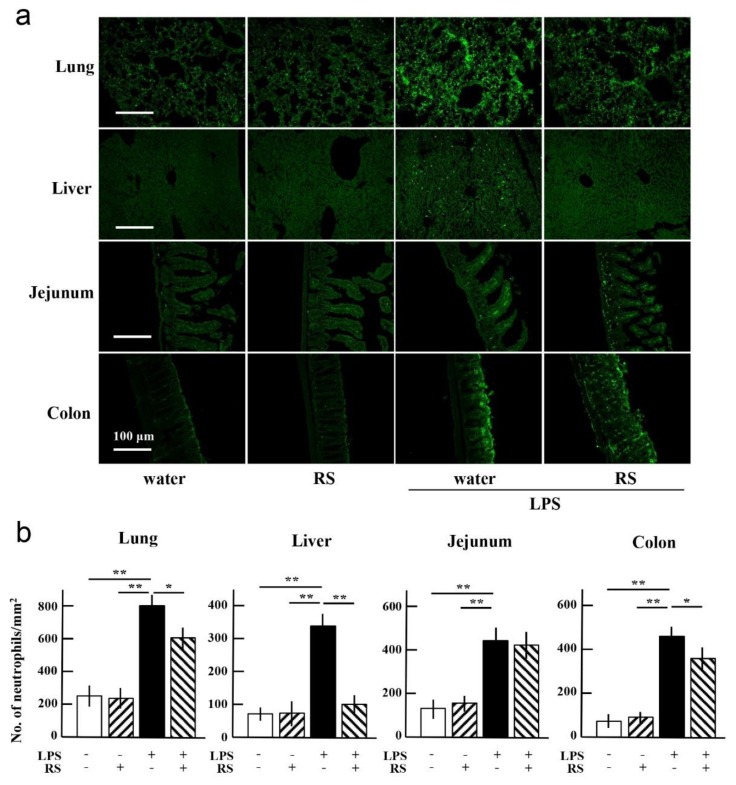
Effects of oral daily administration of RS (1 mg) or water on neutrophil infiltration in organ tissues of mice 24 h after treatment with LPS (50 μg) or saline. (**a**) Immunohistochemical analysis of Gr-1 positive cells in the lung, liver, jejunum, and colon of mice in the water group (RS: -, LPS: -), RS group (RS: +, LPS: -), water + LPS group (RS: -, LPS: +), and RS+LPS group (RS: +, LPS: +). Scale bars: 100 μm. (**b**) Neutrophil infiltration levels in each organ tissue (number of Gr-1-positive cells/mm^2^ tissue area). The data are shown as mean ± SD. * *p* < 0.05 and ** *p* < 0.01 indicate significant differences between the respective groups by Tukey’s multiple comparisons test (*n* = 5).

Histopathological analysis of HE-stained sections from mouse lungs showed that LPS treatment induced inflammatory cell infiltration in the lungs. Prior oral RS administration suppressed cell infiltration, and the effect of RS on neutrophil infiltration into the lungs, liver, jejunum, and colon was investigated. The effect of RS was analyzed using immunochemical staining of tissue sections with an antibody against the neutrophil marker protein Gr-1. As shown in Figure 5a, neutrophil infiltration was increased in all organs of the water + LPS group, especially in the lungs and colon. The increase in neutrophil infiltration due to LPS was significantly suppressed by prior oral administration of RS to the liver (*p* < 0.01) and lungs and colon (*p* < 0.05) but not the jejunum (Figure 5b). These findings indicate that RS suppresses LPS-induced inflammation by inhibiting the infiltration of neutrophils and possibly monocytes/macrophages into the lungs, liver, and colon.

### 2.5. Effect of RS on LPS-Induced Expression of Inflammatory Factors in Organs

**Figure 6:**
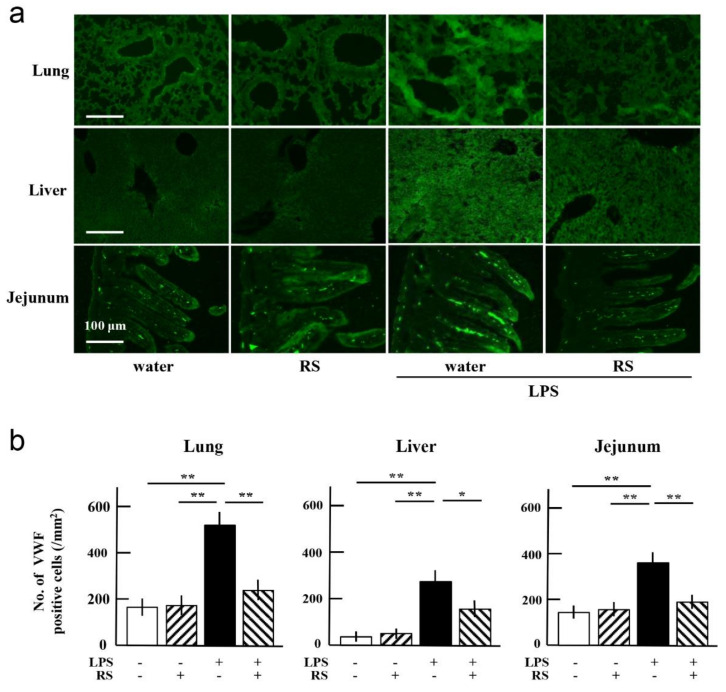
Effects of oral daily administration of RS (1 mg) or water on von Willebrand factor (VWF) expression in organ tissues of mice 24 h after treatment with LPS (50 μg) or saline. (**a**) Immunohistochemical analysis of VWF-positive cells in the tissues of lung, liver, and jejunum of mice in the water group (RS: -, LPS: -), RS group (RS: +, LPS: -), water + LPS group (RS: -, LPS: +), and RS + LPS group (RS: +, LPS: +). Scale bars: 100 μm. (**b**) VWF-positive cells in each organ tissue (number of VWF-positive cells/mm^2^ tissue area). The data are shown as mean ± SD. * *p* < 0.05 and ** *p* < 0.01 indicate significant differences between the respective groups by Tukey’s multiple comparisons test (*n* = 5).

LPS stimulates the expression of inflammatory cytokines such as IL-6, TNF-α, and IL-1β, which increase the expression of VWF and TF in the vascular endothelial cells of organs, and these factors induce platelet aggregation and blood coagulation, respectively [19]. In acute inflammation, such as that caused by accidental injury, and chronic inflammation, such as that caused by metabolic syndrome and cancer, vascular endothelial cells express pro-inflammatory and thrombus-promoting factors such as VWF, TF, and IL-6. Therefore, the effect of RS on LPS-induced expression of VWF, TF, and IL-6 in the lungs, liver, and jejunum of mice was investigated by immunohistochemical analysis and Western blotting (Figure 6). 

**Figure 7:**
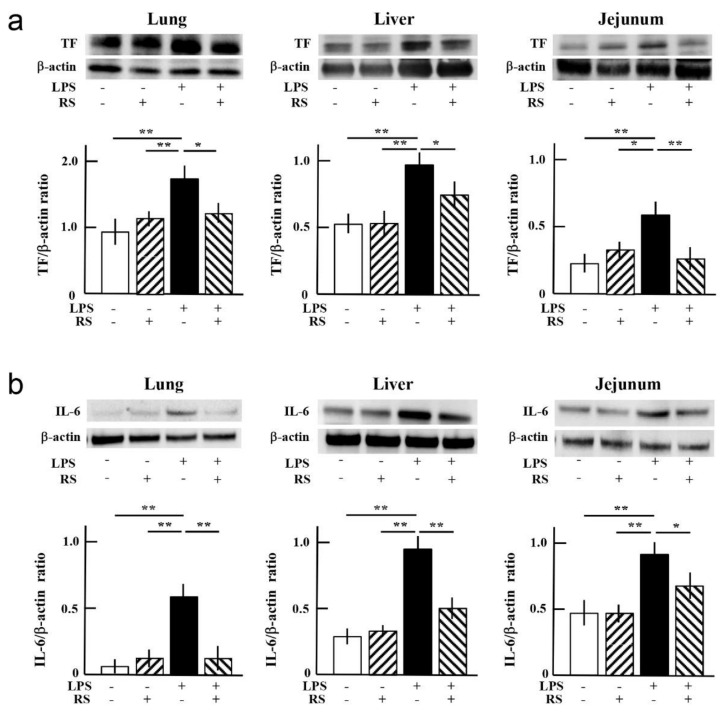
Effects of oral daily administration of RS (1 mg) or water on tissue factor (TF) and interleukin (IL)-6 expression in organ tissues of mice 24 h after treatment with LPS (50 μg) or saline. Representative data from Western blotting regarding expression levels of (**a**) TF and (**b**) IL-6 in the lung, liver, and jejunum of mice in the water group (RS: -, LPS: -), RS group (RS: +, LPS: -), water + LPS group (RS: -, LPS: +), and RS + LPS group (RS: +, LPS: +). Quantification of TF and IL-6 band intensities was normalized to the ratio of β-actin band intensities. The data are shown as mean ± SD. * *p* < 0.05 and ** *p* < 0.01 indicate significant differences between the respective groups by Tukey’s multiple comparisons test (*n* = 3).

Immunohistochemical analysis revealed that the number of VWF-expressing cells was significantly increased in the mouse lungs, liver, and jejunum of the water + LPS group compared to the water group or RS group. The increase in VWF expression was suppressed in all organs of the RS+LPS group, especially in the lungs and jejunum, compared to the water + LPS group (Figure 6a,b). Western blotting analysis showed that TF and IL-6 expression were significantly increased in all organs of the water + LPS group compared to the water or RS group. Increased levels of TF due to LPS treatment were significantly suppressed in all organs, especially in the jejunum of the RS + LPS group, and increased levels of IL-6 due to LPS treatment were significantly suppressed in all organs, especially in the lungs and liver of the RS + LPS group (Figure 7). The data indicate that RS significantly suppresses LPS-induced organ inflammation by inhibiting the expression of pro-inflammatory and thrombus-promoting factors.

### 2.6. Effect of RS on LPS-Induced Expression of Adhesion Molecules in Organs

**Figure 8:**
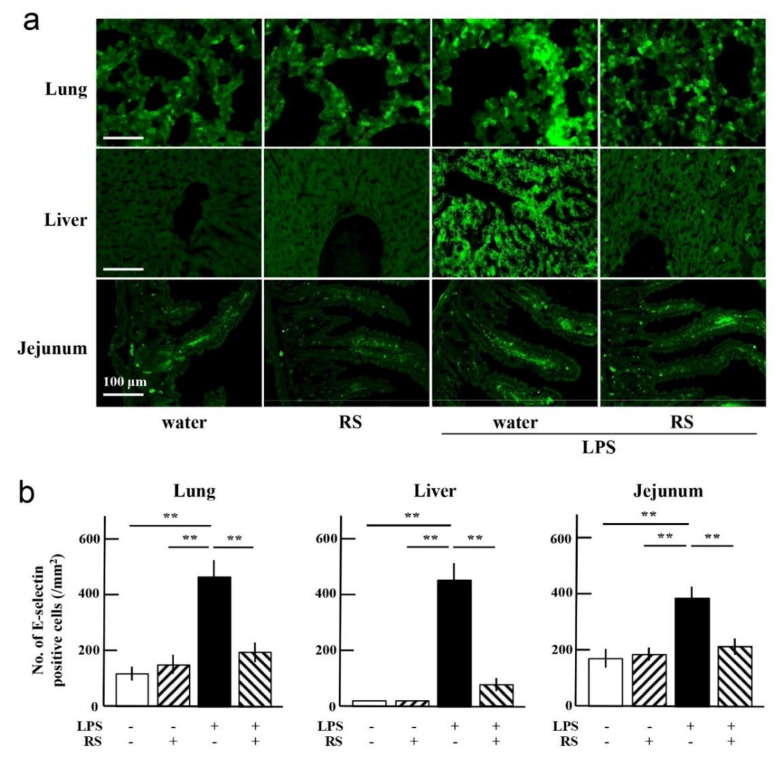
Effects of oral daily administration of RS (1 mg) or water on E-selectin expression in organ tissues of mice 24 h after treatment with LPS (50 μg) or saline. (**a**) Immunohistochemical analysis of E-selectin-positive cells in the lung, liver, and jejunum in the water group (RS: -, LPS: -), RS group (RS: +, LPS: -), water + LPS group (RS: -, LPS: +), and RS + LPS group (RS: +, LPS: +). Scale bars: 100 μm. (**b**) E-selectin-positive cells in tissue of each organ (number of E-selectin-positive cells/mm^2^ tissue area). The data are shown as mean ± SD. * *p* < 0.05 and ** *p* < 0.01 indicate significant differences between the respective groups by Tukey’s multiple comparisons test (*n* = 5).

LPS also stimulates innate immunity-related cells such as monocytes, macrophages, and neutrophils. These immune cells express inflammatory mediators, which enhance inflammatory responses involving leukocyte adhesion by expressing various adhesion molecules such as E-selectin and VCAM-1. E-selectin induces leukocyte rolling and tethering to endothelial cells. This phenomenon is known as the initial step in leukocyte binding to endothelial cells. VCAM-1 then supports the establishment of firm adhesion and transmigration of leukocytes into the subendothelial spaces of the vessel wall. As noted above, LPS stimulated neutrophil infiltration into all examined organs, and orally administered RS suppressed the neutrophil infiltration into the organs (Figure 8). 

**Figure 9:**
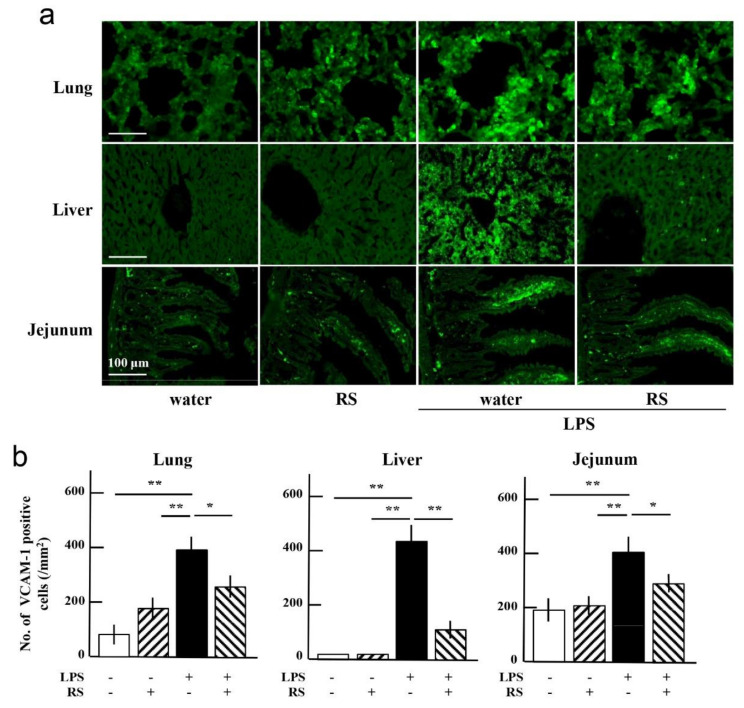
Effects of oral daily administration of RS (1 mg) or water on vascular cell adhesion molecule (VCAM-1) expression in organ tissues of mice 24 h after treatment with LPS (50 μg) or saline. (**a**) Immunohistochemical analysis of VCAM-1-positive cells in the lung, liver, and jejunum in the water group (RS: -, LPS: -), RS (1 mg) group (RS: +, LPS: -), water + LPS group (RS: -, LPS: +), and RS + LPS group (RS: +, LPS: +). Scale bars: 100 μm. (**b**) VCAM-1-positive cells in each organ tissue (number of VCAM-1-positive cells/mm^2^ tissue area). The data are shown as mean ± SD. * *p* < 0.05 and ** *p* < 0.01 indicate significant differences between the respective groups by Tukey’s multiple comparisons test (*n* = 5).

Therefore, to clarify whether RS suppresses the expression of adhesion molecules E-selectin and VCAM-1, which can be induced by LPS treatment, the level of expression of these molecules in the tissues of the lungs, liver, and jejunum was determined using immunohistochemical analysis. As shown in Figure 8a,b, E-selectin-expressing cells were increased in all mouse organs of the water + LPS group compared to the water or RS group. The increased levels of E-selectin due to LPS treatment were significantly suppressed in all organs of the RS + LPS group, especially in the liver. VCAM-1-expressing cells were also increased in all organs of the water + LPS group, and the increased levels of VCAM-1 were significantly suppressed in all organs of the RS+LPS group, especially in the liver (Figure 9a,b). These results indicate that daily administration of RS prior to LPS treatment suppresses the expression of adhesion molecules on the endothelium during inflammation and attenuates leukocyte infiltration into the organs (Figure 9).

### 2.7. Effect of RS on LPS-Induced Degradation of Glycocalyx and Syndecan-4

**Figure 10:**
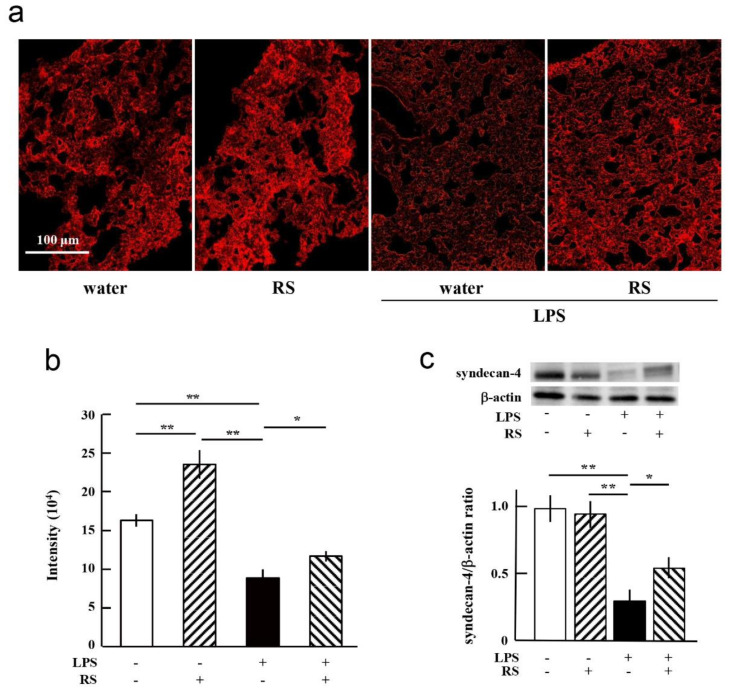
Effects of oral daily administration of RS (1 mg) or water on glycocalyx levels in lung tissue of mice 24 h after treatment with LPS (50 μg) or saline. (**a**) Wheat germ agglutinin (WGA) staining of glycocalyx in the lung tissue of mice in the water group (RS: -, LPS: -), RS group (RS: +, LPS: -), water + LPS group (RS: -, LPS: +), and RS + LPS group (RS: +, LPS: +). Glycocalyx in the lung tissue was stained using WGA followed by fluorescence detection. Scale bars: 100 μm. (**b**) Fluorescence intensity levels of WGA-stained lung tissues of mice in each group. The data are shown as mean ± SD. * *p* < 0.05 and ** *p* < 0.01 indicate significant differences between the respective groups by Tukey’s multiple comparisons test (*n* = 5). (**c**) Representative data of Western blotting and expression levels of syndecan-4 in the lung tissues of mice in each group. Quantification of syndecan-4 band intensities was normalized to the ratio of β-actin band intensities. The data are shown as mean ± SD. * *p* < 0.05 and ** *p* < 0.01 indicate significant differences between the respective groups by Tukey’s multiple comparisons test (*n* = 3).

In LPS-induced sepsis, the expression of pro-inflammatory markers, such as IL-6 and TNF-α, is associated with the degradation of the vascular endothelium glycocalyx [35]. Therefore, we hypothesized that RS may protect against endothelial glycocalyx degradation induced by LPS treatment in mice. To test this, changes in endothelial glycocalyx were determined using wheat germ agglutinin (WGA) binding in lung sections of mice treated with LPS with or without prior oral administration of RS. As shown in Figure 10a,b, the levels of WGA bound to the lung tissue in the RS group were significantly (*p* < 0.01) increased compared to those in the water group (control). In contrast, the level of WGA binding in the lungs of the water + LPS group was significantly (*p* < 0.01) reduced compared to both the water group and the RS group. In addition, the level of WGA binding in the lungs of the RS+LPS group was significantly (*p* < 0.05) increased compared to that of the water + LPS group. These results indicate that orally administered RS increases vascular endothelium glycocalyx levels and protects the vascular endothelial glycocalyx from LPS-induced degradation.

To investigate the mechanism of protection by RS against the degradation of glycocalyx by LPS in more detail, the amount of syndecan-4 was determined using Western blotting. As shown in Figure 10c, levels of syndecan-4 were significantly (*p* < 0.01) reduced in the mouse lungs of the water + LPS group compared to those in the water or RS group. Syndecan-4 levels were significantly (*p* < 0.05) increased in the lungs of the RS + LPS group compared to the water + LPS group. The data show that LPS treatment induced the degradation of endothelial syndecan-4 and that LPS-induced degradation of syndecan-4 was prevented by prior oral administration of RS.

## 3. Discussion

Previously, we found that RS purified from the extract of *M. nitidum* inhibits thrombin in the presence of antithrombin, like heparin, and RS, unlike heparin, suppresses inflammation of cultured human umbilical vein endothelial cells (HUVECs) induced by LPS, TNF-α, or thrombin [8]. In the present study, we investigated the effect of oral administration of RS on LPS-induced inflammation in mouse organs and vascular endothelium to determine whether RS exhibits anti-inflammatory effects in vivo.

The present study data show that prior oral administration of RS suppresses LPS-induced increases in pulmonary vascular permeability and hepatocellular injury in mice. In addition, oral administration of RS suppressed morphological and functional damage in the lungs, liver, jejunum, and colon caused by LPS-induced inflammation. In addition, orally administered RS suppressed the expression of pro-inflammatory and thrombus-promoting factors VWF, TF, and IL-6. It further suppressed the expression of adhesion molecules E-selectin and VCAM-1, which are required for leukocyte adhesion on the endothelium and infiltration into the organs. These findings indicate that RS suppresses organ damage by inhibiting the infiltration of neutrophils and possibly monocytes/macrophages into the organs. In addition, orally administered RS increased vascular endothelium glycocalyx levels and protected vascular endothelial glycocalyx from LPS-induced degradation. Oral RS administration also suppressed LPS-induced degradation of syndecan-4, a type of glycocalyx, on the vascular endothelium. These findings suggest that oral administration of RS prevents LPS-induced inflammatory disorders of vascular endothelial cells that cause inflammatory damage to organs. However, the scientific basis for how RS works is not fully understood. In particular, there are unclear points about the mechanism by which RS suppresses inflammatory disorders, such as glycocalyx loss of vascular endothelial cells, and the mechanism by which orally administered RS is taken up into blood vessels from the lumen of the small intestine.

The mechanism by which RS suppresses inflammatory disorders has been described in previous studies and is as follows. The glycocalyx layer is extremely fragile and sheds from the vascular endothelium due to a variety of factors [25,29,36]. Glycocalyx shedding occurs due to ischemia–reperfusion injury; hypervolemia; sepsis; malaria; chronic diseases such as hyperglycemia, arteriosclerosis, and diabetes [26,37,38,39]; inflammatory cytokines; sialidase treatment; and administration of sialyltransferase inhibitors [40]. Destruction of the glycocalyx layer causes a series of leaks from capillaries and the edema, the promotion of inflammatory reactions, and the adhesion of platelets to the wall of blood vessels [27,28]. At the experimental level, compounds such as glucocorticoids, antithrombin, TNF-α inhibitors, and antioxidants such as allopurinol have been reported to be effective in protecting the glycocalyx [38,41]. The administration of sulfated polysaccharides such as heparan sulfate extracted from the glycocalyx to mice suppresses the binding of leukocytes to the endothelium and the onset of arteriosclerosis due to oxidized low-density lipoprotein [42]. The treatment of cultured endothelial cells with heparan sulfate, the carbohydrate component of syndecan 4, and sphingosine-1-phosphate, a metabolite of lipids that makes up biological membranes, promotes glycocalyx regeneration and restoration of inter-endothelial communication [43]. Sialic acid, a component of the glycocalyx, plays an important role in antioxidant activity and is catalyzed by sialyltransferase, an enzyme that transfers sialic acid to nascent oligosaccharides. Sevoflurane, used as an inhalational anesthetic for the induction and maintenance of general anesthesia, significantly upregulates the expression of sialyltransferase, promotes the recovery of endothelial glycocalyx, and enhances endothelium-dependent vasodilation after oxidative stress [40]. In this study, as shown in Figure 10b, orally administered RS increased vascular endothelial glycocalyx levels and protected vascular endothelial glycocalyx from LPS-induced degradation in mice. Previously, we reported that RS reduced inflammatory damage in cultured HUVECs [8]. RS significantly suppressed the increase in TF and VWF expression levels in both LPS-stimulated HUVECs and HUVECs not stimulated with LPS. Unlike RS, heparin enhanced VWF expression in HUVECs both in the presence and absence of LPS. In addition, RS significantly suppressed elevated TF and VWF expression in TNF-α or thrombin-induced HUVEC inflammation, but heparin did not show such a suppressive effect. Therefore, RS directly protects vascular endothelial cells from inflammatory stimuli, presumably by stabilizing the glycocalyx-containing syndecan-4 from degradation by sialidase or by increasing the expression of sialyltransferase in vascular endothelial cells. Further research is needed to elucidate the exact mechanisms underlying the protective effect of RS on endothelial cells. 

On the other hand, the mechanism by which orally administered RS is taken up from the intestine into blood vessels is speculated as follows. RS is a sulfated rhamnose polymer with a molecular weight ranging from 50,000 to 1,000,000 Da. Therefore, its direct absorption into the blood after ingestion seems difficult. A substance similar to RS is fucoidan, a dietary fiber that is abundant in the mucus of brown algae such as kelp, wakame seaweed, and mozuku seaweed. Fucoidan is a type of sulfated polysaccharide with an average molecular weight of about 200,000 made of fucose polymer. It has been reported that fucoidan was detected in serum at a concentration of about 50 ng/ml 6 h after a single oral dose of 1 g of fucoidan to humans [44]. This suggests that small amounts of fucoidan can be absorbed into the blood from the intestines. Studies in rats have shown that fucoidan is absorbed from the intestinal epithelium and transported to the liver [45]. Therefore, it is predicted that RS, which has a structure similar to fucoidan, may also be absorbed into the blood by this mechanism. Furthermore, it is known that M cells in the epithelial cells of the intestine selectively absorb macromolecules and microorganisms into the body. We recently reported that fluorescein isothiocyanate-labeled RS co-localizes with M cells after oral administration to mice [7]. These facts suggest that RS is absorbed into the blood via intestinal M cells after oral ingestion and has an anti-inflammatory effect on the vascular endothelium. 

In addition, in patients with COVID-19, a new coronavirus infection, inflammatory disorders of the vascular endothelium are known to be associated with exacerbation of symptoms, similar to sepsis. Therefore, protection of vascular endothelium glycocalyx may be a limiting factor for COVID-19 [46]. RS may be an excellent candidate for the control of various diseases/disorders caused by the breakdown of the glycocalyx.

## 4. Materials and Methods

### 4.1. Preparation of RS

RS was purified from the hot water extract of *M. nitidum* using a previously described method [7]. The purified RS (Konan Chemical Manufacturing Co., Ltd., Yokkaichi, Mie, Japan; purity: 94%) showed a single peak with a shoulder in front of the main peak (average molecular weight: 5 × 10^5^ Da) in gel permeation chromatography. 

### 4.2. Animal Experiments

Specific-pathogen-free (SPF), 10-week-old BALB/c male mice (SLC, Hamamatsu, Shizuoka, Japan) were used. They were housed in individual cages in an air-conditioned room at 23 ± 1 °C under SPF conditions with a 12 h light–dark cycle. 

To evaluate the effect of RS on LPS-induced lung vascular permeability, three groups of mice were created, as shown in Figure 2a. Water or different doses of RS (0.25, 0.5, 1, or 2.5 mg) were orally administered by feeding needle daily to mice for 8 d, and 30 min after the last water or RS administration, 50 μg of LPS (MilliporeSigma, Burlington, MA, USA) was intraperitoneally administered, creating the water + LPS group (*n* = 3) and RS+LPS group (*n* = 3). For the control, water was administered for 8 d, and 100 μL of saline was intraperitoneally administered. This was the water group (*n* = 3). Six hours post-LPS or post-saline treatment, EB dye was injected into the tail vein of mice, and 30 min later, the lungs were resected from each mouse. The lungs were dried at 65 ℃ overnight in an oven and then added to 500 µL of formamide solution. Lung samples were then incubated at 60 ℃ for 24 h to extract the EB dye. Following this, the absorbance of the solution at 620 nm was measured, and the amount of extravasated EB (µg/mL/mg tissue) was calculated. 

To examine the effect of RS on LPS-induced morphological and functional changes in the organ tissues of mice, four groups were prepared as shown in Figure 3a. Water or 1 mg of RS was orally administered daily to mice for 8 d, and 30 min after the last water or RS administration, the mice were intraperitoneally treated with saline (water group, *n* = 5; RS group, *n* = 5) or 50 μg of LPS (water + LPS group, *n* = 5; and RS + LPS group, *n* = 5). Twenty-four hours later, heparinized blood was obtained from the heart of each mouse under anesthesia, and the organs were resected from the mice.

All studies using mice were conducted in accordance with the recommendations of the Guide for the Care and Use of Laboratory Animals of Suzuka University of Medical Science (approval number: 63). All surgeries were performed under pentobarbital anesthesia, and maximum effort was taken to minimize animal suffering.

### 4.3. Measurement of Liver Deviation Enzymes in Mouse Plasma

Plasma levels of molecular markers of liver damage (CK, GOT, and GPT) in mice were measured using commercial kits (CK: BioAssay Systems, Hayward, CA, USA; GOT: Wako, Osaka, Japan; GPT: Wako, Osaka, Japan) according to the manufacturers’ instructions.

### 4.4. Preparation of Organ Sample Specimens

The organ samples were fixed in 4% phosphate-buffered paraformaldehyde, embedded in frozen Tissue Tek, OCT compound (Sakura Finetek, Tokyo, Japan), and cut into 5 μm thick sections. These sections were subjected to HE staining in accordance with the established procedures for histological analysis of the lung, liver, jejunum, and colon. Thereafter, the specimens were stained using antibodies for immunohistological analysis, as described previously [47]. The lung, liver, and jejunum specimens were incubated with rat monoclonal Gr-1 (Ly6G, a marker of neutrophils; 1:100; R&D Systems, Minneapolis, MN, USA), rabbit polyclonal E-selectin (1:100; Bioss, Woburn, MA, USA), rabbit polyclonal VCAM-1 (1:100; Abcam, Cambridge, MA, USA), or rabbit monoclonal VWF (1:100; Abcam) primary antibodies. The specimens were subsequently incubated with fluorescein isothiocyanate-conjugated anti-rat and anti-rabbit secondary antibodies (1:30; DakoCytomation, Glastrup, Denmark). The expression of neutrophils, E-selectin, VCAM-1, and VWF was evaluated immunohistochemically using fluorescence microscopy. Neutrophils (Gr-1-positive cells) and E-selectin-, VCAM-1-, or VWF-expressing cells were determined by laser scanning confocal microscopy using a Bio-Rad MRC500 instrument (Bio-Rad Laboratories Inc., Hercules, CA, USA) mounted on an Olympus microscope (Olympus, Tokyo, Japan). The number of neutrophils and E-selectin-, VCAM-1-, and VWF-positive cells were quantified based on image intensity using ImageJ software. For analysis of glycocalyx injury, the lung specimens were incubated with WGA, Triticum vulgaris, and biotin conjugate (1:250; Vector Laboratories, Burlingame, CA, USA). The specimens were subsequently incubated with streptavidin DyLight^®^594 (1:200; VECTOR, Burlingame, CA, USA).

### 4.5. Western Blot Analysis of the Lung, Liver, and Jejunum

The organ samples were homogenized in lysis buffer (Kurabo, Osaka, Japan) and centrifuged at 8000× *g* for 10 min. Western blot analysis was performed as described previously [48]. The membranes were incubated at room temperature for 1 h with primary antibodies against TF (1:1000; Bioss, Woburn, MA, USA), syndecan-4 (1:1000; LifeSpan BioSciences, Seattle, WA, USA), IL-6 (1:1000; R&D Systems, Minneapolis, MN, USA), or β-actin as a loading control (1:5000; Sigma-Aldrich, St. Louis, MO, USA). Thereafter, the membranes were washed and incubated with a horseradish-peroxidase-conjugated secondary antibody (Novex, Frederick, MD, USA). The immune complexes were detected using ImmunoStar Zata reagent (Wako, Osaka, Japan), and images were acquired using the Multi Gauge software program (Fujifilm, Greenwood, SC, USA).

### 4.6. Statistical Analysis

All data are expressed as mean ± standard deviation. Results were analyzed using Microsoft Excel 2010. Multiple comparisons between groups were calculated by one-way analysis of variance followed by Tukey’s post hoc test using SPSS version 20 (IBM, Armonk, NY, USA). The differences were considered statistically significant at *p* < 0.05.

## Data Availability

The data presented in this study are available on request from the corresponding author.
